# MaGIC: a machine learning tool set and web application for monoallelic gene inference from chromatin

**DOI:** 10.1186/s12859-019-2679-7

**Published:** 2019-02-28

**Authors:** Svetlana Vinogradova, Sachit D. Saksena, Henry N. Ward, Sébastien Vigneau, Alexander A. Gimelbrant

**Affiliations:** 1 Department of Cancer Biology, Dana-Farber Cancer Institute, Boston, MA 02115 USA; 2 Department of Genetics, Harvard Medical School, Boston, MA 02115 USA; 3 University of Minnesota-Twin Cities, Bioinformatics and Computational Biology Program, Minneapolis, MN 55455 USA; 4 Computational and Systems Biology, Massachusetts Institute of Technology, Cambridge, MA 02139 USA

**Keywords:** Monoallelic expression, Chromatin, Chromatin signature, Software pipeline, Shiny app

## Abstract

**Background:**

A large fraction of human and mouse autosomal genes are subject to random monoallelic expression (MAE), an epigenetic mechanism characterized by allele-specific gene expression that varies between clonal cell lineages. MAE is highly cell-type specific and mapping it in a large number of cell and tissue types can provide insight into its biological function. Its detection, however, remains challenging.

**Results:**

We previously reported that a sequence-independent chromatin signature identifies, with high sensitivity and specificity, genes subject to MAE in multiple tissue types using readily available ChIP-seq data. Here we present an implementation of this method as a user-friendly, open-source software pipeline for **m**ono**a**llelic **g**ene **i**nference from **c**hromatin (MaGIC). The source code for the MaGIC pipeline and the Shiny app is available at https://github.com/gimelbrantlab/magic.

**Conclusion:**

The pipeline can be used by researchers to map monoallelic expression in a variety of cell types using existing models and to train new models with additional sets of chromatin marks.

**Electronic supplementary material:**

The online version of this article (10.1186/s12859-019-2679-7) contains supplementary material, which is available to authorized users.

## Background

Genotype-phenotype relationship in mammals is profoundly affected by three epigenetic phenomena that control the relative expression of the two parental alleles: imprinting, X-chromosome inactivation, and autosomal random monoallelic expression (MAE) [1, 14]. MAE is the most widespread of these three phenomena, affecting over 10% of human autosomal genes, including multiple genes implicated in cancer, autism, and Alzheimer’s disease [5, 6, 13]. Similar to X-inactivation, the active allele is randomly selected early in the development and then maintained in mitotically stable manner, making MAE clone-specific [2, 4, 17]. As a result, existing allelic imbalances at the level of individual clones cannot be detected in polyclonal, tissue-level sequencing experiments. As an alternative to direct measurement, we have identified a chromatin signature of monoallelic expression, which can be applied to detect MAE in polyclonal samples [10].

This chromatin signature of MAE is based on gene-body enrichment of histone H3 Lys-27 trimethylation (H3K27me3) and H3 Lys-36 trimethylation (H3K36me3) as measured by ChIP-seq. The first chromatin mark is associated with active transcription and the second one is associated with silencing; MAE genes are enriched among genes displaying a characteristic chromatin signature: the two marks simultaneously occurring in the gene body. We experimentally confirmed the signature’s accuracy in multiple human and mouse cell-types using clonal cell lines with known allelic expression as a reference [10, 11]. Use of the chromatin-based MAE maps has already led to new insight in genome evolution [15] and neurodevelopmental disease [16]. However, the initial implementation of the method was not integrated in a unified, shareable pipeline and had limited flexibility. We thus set out to build a user-friendly, flexible, open-source toolset to enable systematic analysis of a variety of large-scale datasets.

Here, we describe an R pipeline named **m**ono**a**llelic **g**ene **i**nference from **c**hromatin (MaGIC) with command-line and Shiny app interface. In addition to classifying genes as MAE or biallelic based on existing models, MaGIC can also generate predictive models de novo as new, larger datasets become available.

## Implementation

**Fig. 1:**
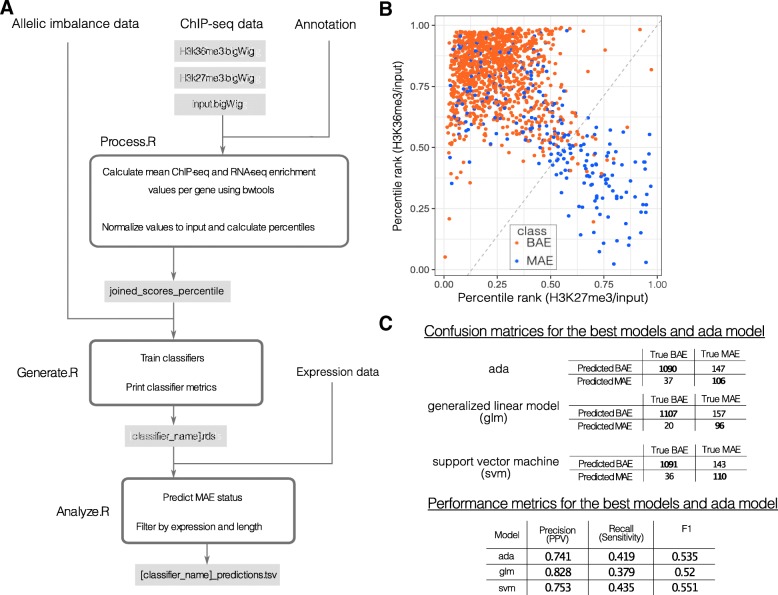
The MaGIC 2.0 pipeline and evaluation of glm performance. **a** *Process.R* calculates ChIP-seq enrichment per gene from ChIP-seq and control bigWig files. *Generate.R* trains classifiers using ChIP-seq enrichment and true MAE/BAE calls. *Analyze.R* uses classifiers to predict MAE/BAE gene status from ChIP-seq data. **b** Chromatin signature space of classifier features (H3K27me3 vs. H3K36me3) with the glm’s decision boundary plotted (*dashed line*) with MAE status of true labeled testing data (MAE: *blue*, BAE: *red*). **c** Confusion matrices and performance metrics for the selected models evaluated on additional testing data (glm and svm are selected as the best models based on the precision and the recall; ada model is presented for comparison reasons as the closest to the model in [11])

MaGIC is a command-line software package written in R that consists of three separate parts (Fig. 1a).

**Process.R** uses ChIP-seq bigWig [7] files to calculate gene-body or promoter enrichment normalized to control data (e.g. ChIP input) or to feature length. First, Bwtool [12] is used to calculate mean signals for gene intervals based on a reference annotation. X-linked, imprinted, and olfactory receptor genes are then filtered out by default to focus on the less characterized autosomal random MAE genes. Intervals with control signals lower than a user-defined threshold are also removed. Finally, ChIP-seq signal is normalized to control signal, and the resulting values are converted to quantile rank and saved to a file. This output file can be used to generate new classifiers using *generate.R* or to predict monoallelic expression using *analyze.R* with pre-trained classifiers.

**Generate.R** trains classifiers on ChIP-seq data processed by the *process.R* script using a variety of algorithms supported by the *caret* R package [8]. A set of training labels containing true allelic expression calls for genes in the same tissue should be provided in a separate file. Alternatively, the user can use one of the training label sets we provide, which list MAE and BAE genes in human and mouse B lymphoid cells. By default, *generate.R* trains models with five-fold cross-validation, although the degree of cross-validation can be modified by the user. If the user has a separate validation set, they can instead train the classifier on the complete set of training data. In all cases, *generate.R* outputs a set of models and a summary file containing per-model performance metrics. By default, a total of 9 models are trained, including a neural network with two hidden layers, a support vector machine with multiple kernels, a multi-layer perceptron, three tree-based models (an adaptive boosted classification tree - ada, tree models from genetic algorithms, and recursive partitioning and regression trees), random forest, a K-Nearest Neighbor model and a generalized linear model with stepwise feature selection (glm). Among all models trained, we recommend choosing the model with the highest F1-score as discussed below. Therefore, this model is selected by default for subsequent analysis but the user has the option to override this choice by selecting additional or different models.

**Analyze.R** predicts monoallelic expression using ChIP-seq data processed by *process.R* and classifiers generated by *generate.R*. The predictions can be filtered by minimal gene length and expression level, with values provided by the user in separate files, with default thresholds as defined in [11]. The output from *analyze.R* contains predicted allelic expression status by gene.

### Shiny web application

To make the MaGIC software more user-friendly and add additional visualizations, we developed a web application using the Shiny framework. This graphical user interface offers all the functionality of the pipeline with a streamlined workflow and can be run locally following installation from https://github.com/gimelbrantlab/magic.

## Results

The MaGIC pipeline begins with ChIP-seq data processing and concludes with the prediction of MAE genes based on this data (see Implementation for more details). First, we process ChIP-seq files into gene-body or promoter enrichment normalized to control data. Next, this processed signal is used to classify genes into MAE and BAE using existing or user-generated models. New models can be generated with a training set of genes containing true allelic expression calls, typically determined by RNA-Seq in related clonal cell lines and allowing to directly classify genes as MAE or BAE.

### Software validation

In order to validate MaGIC software, we trained a monoallelic expression classifier using the same datasets as in our previous studies [10, 11]. The datasets include ChIP-seq H3K27me3 and H3K36me3 enrichment data for the GM12878 human B-lymphoblastoid cell line [3] and a list of 263 monoallelically expressed and 1024 biallelically expressed genes identified in human B-lymphoblastoid clonal cell lines [4]. We used precision and recall to assess the classifiers performance, which are defined as the fraction of correct MAE predictions among all genes predicted as MAE and the fraction of correct MAE predictions over the total number of MAE genes in the dataset, respectively.

MAE genes make up between 5 and 20% of expressed genes in a given tissue, so these datasets are naturally imbalanced. In order to avoid excessive numbers of false positive calls due to this imbalance, we trained the models to optimize the metric Kappa rather than accuracy, as Kappa accounts for imbalanced number of genes belonging to each class in training data [9]. We trained a total of 9 models, including a neural network, a support vector machine, a multi-layer perceptron, three tree-based models, random forest, a K-Nearest Neighbor model, and a generalized linear model (glm).

After the training step, all models were tested on an additional human dataset with 253 MAE genes and 1127 BAE genes identified in monoclonal cell lines derived from GM12878 (Dataset S2 from [10]).

Among all models tested, glm had the highest precision value and svm had the highest F1 score (Fig. 1b, c; Additional file 1: Table S1). The choice between models can be made using one of the performance metrics, depending on the purpose of the analysis. We generally recommend to use the F1 score, which is a balanced metric calculated as a harmonic mean of precision and recall. The precision score is superior if the user wants to have the lowest number of false positives possible e.g., in identifying high-confidence MAE genes. These gene lists can be further used to design experiments aimed at studying MAE genes’ properties. However, it should be noted that high precision values come at the expense of recall or general coverage of the dataset as the classifier misses a significant portion of MAE genes in the sample via false negative predictions. The F1 score is useful if the user is performing genome-wide analysis and wants a higher coverage of MAE genes. In both cases, further experimental validation is recommended, but the initial lists of MAE genes are a good starting point for guiding experimental design and exploratory analysis.

The generalized linear model is packaged along with the MaGIC software. It was also evaluated on mouse B-lymphoid clonal cell lines, mouse fibroblasts and mouse neural progenitor cells (data from Nag et al., 2015 [11], Tables S2 and Table S3 and it performed similarly to the classifier from Nag et al., 2015 [11] (Additional file 2: Table S2). The classifier performance on the mouse datasets tested is still low compared to the human datasets (the precision is 0.56–0.65 and the recall is 0.08–0.45,depending on the cell type), but this may be caused by discrepancy in the quality of the data rather than profound differences in MAE chromatin signature between the two species, as previously discussed [11]. In particular, due to the limited number of clones assessed and differences originating in the derivation process of the F1 genetic background, the GLM’s performance metrics provide a lower-bound estimate of the potential accuracy in polyclonal cell populations. Using more histone marks data originating from high-quality ChIP-seq experiments and getting matching training data from a bigger number of clones would potentially increase the performance of the classifiers and allow for more precise predictions of MAE genes in the mouse.

## Conclusion

The MaGIC toolset builds on our previously reported MAE chromatin signature classifier in two important ways. It enhances the previously published method using open source tools in a platform-independent running environment with clear documentation. Additionally, the new toolset can generate models using new data and automatically assess the models’ performance. As epigenomic data is becoming increasingly available in many cell and tissue types, we believe the versatility of the MaGIC toolset will prove invaluable to investigate MAE’s mechanisms, function, and contribution to disease.

## Availability and requirements

**Project home page:** https://github.com/gimelbrantlab/magic

**Operating system(s):** Platform independent, browser-based.

**Programming language:** R.

**Other requirements:** Modern web browser, Docker if ran as a Docker container.

**License:** MIT License.

**Any restrictions to use by non-academics:** none.

## Additional files

Additional file 1 **Table S1.** Models’ performances evaluated on an additional human dataset, with MAE and BAE genes identified in monoclonal cell lines derived from GM12878 ([10], Dataset S2). (PDF 26 kb)

Additional file 2 **Table S2.** The generalized linear model performance evaluated on mouse B-lymphoid clonal cell lines (B-lymph), mouse embryonic fibroblasts (MEF) and mouse neural progenitor cells (NPC). (PDF 310 kb)

## References

[1] Chess A (2016). Monoallelic Gene Expression in Mammals. Annu Rev Genet.

[2] Eckersley-Maslin MA, Spector DL (2014). Random monoallelic expression: regulating gene expression one allele at a time. Trends Genet.

[3] ENCODE Project Consortium (2012). An integrated encyclopedia of DNA elements in the human genome. Nature.

[4] Gimelbrant A (2007). Widespread monoallelic expression on human autosomes. Science.

[5] Gui B (2017). Perspective: Is Random Monoallelic Expression a Contributor to Phenotypic Variability of Autosomal Dominant Disorders?. Front Genet.

[6] Ha G (2012). Integrative analysis of genome-wide loss of heterozygosity and monoallelic expression at nucleotide resolution reveals disrupted pathways in triple-negative breast cancer. Genome Res.

[7] Kent WJ (2010). BigWig and BigBed: enabling browsing of large distributed datasets. Bioinformatics.

[8] Kuhn M. Building Predictive Models inRUsing thecaretPackage. J Stat Softw. 2008;28.

[9] Landis JR (1977). The Measurement of Observer Agreement for Categorical Data. Biometrics.

[10] Nag A (2013). Chromatin signature of widespread monoallelic expression. Elife.

[11] Nag A (2015). Chromatin Signature Identifies Monoallelic Gene Expression Across Mammalian Cell Types. G3.

[12] Pohl A, Beato M (2014). bwtool: a tool for bigWig files. Bioinformatics.

[13] Polson ES (2013). Monoallelic expression of TMPRSS2/ERG in prostate cancer stem cells. Nat Commun.

[14] Savova V (2013). Autosomal monoallelic expression: genetics of epigenetic diversity?. Curr Opin Genet Dev.

[15] Savova V (2016). Genes with monoallelic expression contribute disproportionately to genetic diversity in humans. Nat Genet.

[16] Savova V (2017). Risk alleles of genes with monoallelic expression are enriched in gain-of-function variants and depleted in loss-of-function variants for neurodevelopmental disorders. Mol Psychiatry.

[17] Zwemer LM (2012). Autosomal monoallelic expression in the mouse. Genome Biol.

