# Supplementary material for: MaGIC: a machine learning tool set and web application for monoallelic gene inference from chromatin
Source: BMC Bioinformatics. 2019 Feb 28;20:106. doi: 10.1186/s12859-019-2679-7 (PMC6394031; doi:10.1186/s12859-019-2679-7)
Supplement: Supplementary file 1 — Table S1. Models’ performances evaluated on an additional human dataset, with MAE and BAE genes identified in monoclonal cell lines derived from GM12878 ([10], Dataset S2). (PDF 26 kb) [file 12859_2019_2679_MOESM1_ESM.pdf]

Table S1. Models' performances evaluated on an additional human dataset, with MAE and BAE genes identified in monoclonal cell lines derived from GM12878 (Nag et al., 2013, Dataset S2).

Model	Recall (Sensitivity)	Specificity	Precision (PPV)	NPV	F1	Accuracy	Balanced Accuracy
**ada**	0.419	0.967	0.741	0.881	0.535	0.867	0.693
**glmStepAIC**	0.379	0.982	0.828	0.876	0.52	0.872	0.681
**svmPoly**	0.435	0.968	0.753	0.884	0.551	0.87	0.701
**nnet**	0.415	0.972	0.766	0.881	0.538	0.87	0.693
**mlpML**	0.443	0.959	0.709	0.885	0.545	0.864	0.701
**rf**	0.395	0.96	0.69	0.876	0.503	0.857	0.678
**rpart**	0.423	0.955	0.677	0.881	0.521	0.857	0.689
**knn**	0.379	0.97	0.738	0.874	0.501	0.862	0.675
**evtree**	0.423	0.955	0.677	0.881	0.521	0.857	0.689
