# Supplementary material for: MaGIC: a machine learning tool set and web application for monoallelic gene inference from chromatin
Source: BMC Bioinformatics. 2019 Feb 28;20:106. doi: 10.1186/s12859-019-2679-7 (PMC6394031; doi:10.1186/s12859-019-2679-7)
Supplement: Supplementary file 2 — Table S2. The generalized linear model performance evaluated on mouse B-lymphoid clonal cell lines (B-lymph), mouse embryonic fibroblasts (MEF) and mouse neural progenitor cells (NPC). (PDF 310 kb) [file 12859_2019_2679_MOESM2_ESM.pdf]

**Table S2.** The generalized linear model performance evaluated on mouse B-lymphoid clonal cell lines (B-lymph), mouse embryonic fibroblasts (MEF) and mouse neural progenitor cells (NPC).

| Cells | Recall (sensitivity) | Specificity | Precision (PPV) | NPV | Accuracy | Balanced Accuracy |
|---|---|---|---|---|---|---|
| **B-lymph** | 0.094 | 0.961 | 0.564 | 0.665 | 0.659 | 0.527 |
| MEF | 0.084 | 0.981 | 0.65 | 0.721 | 0.718 | 0.533 |
| NPC | 0.453 | 0.898 | 0.586 | 0.837 | 0.79 | 0.675 |
